# Supplementary material for: Enhancing mucosal immunity by transient microbiota depletion
Source: Nat Commun. 2020 Sep 8;11:4475. doi: 10.1038/s41467-020-18248-4 (PMC7479140; doi:10.1038/s41467-020-18248-4)
Supplement: Supplementary file 1 — Supplementary Information [file 41467_2020_18248_MOESM1_ESM.pdf]

Becattini et al., ‘Enhancing Mucosal Immunity by Transient Microbiota Depletion’
Supplementary Information

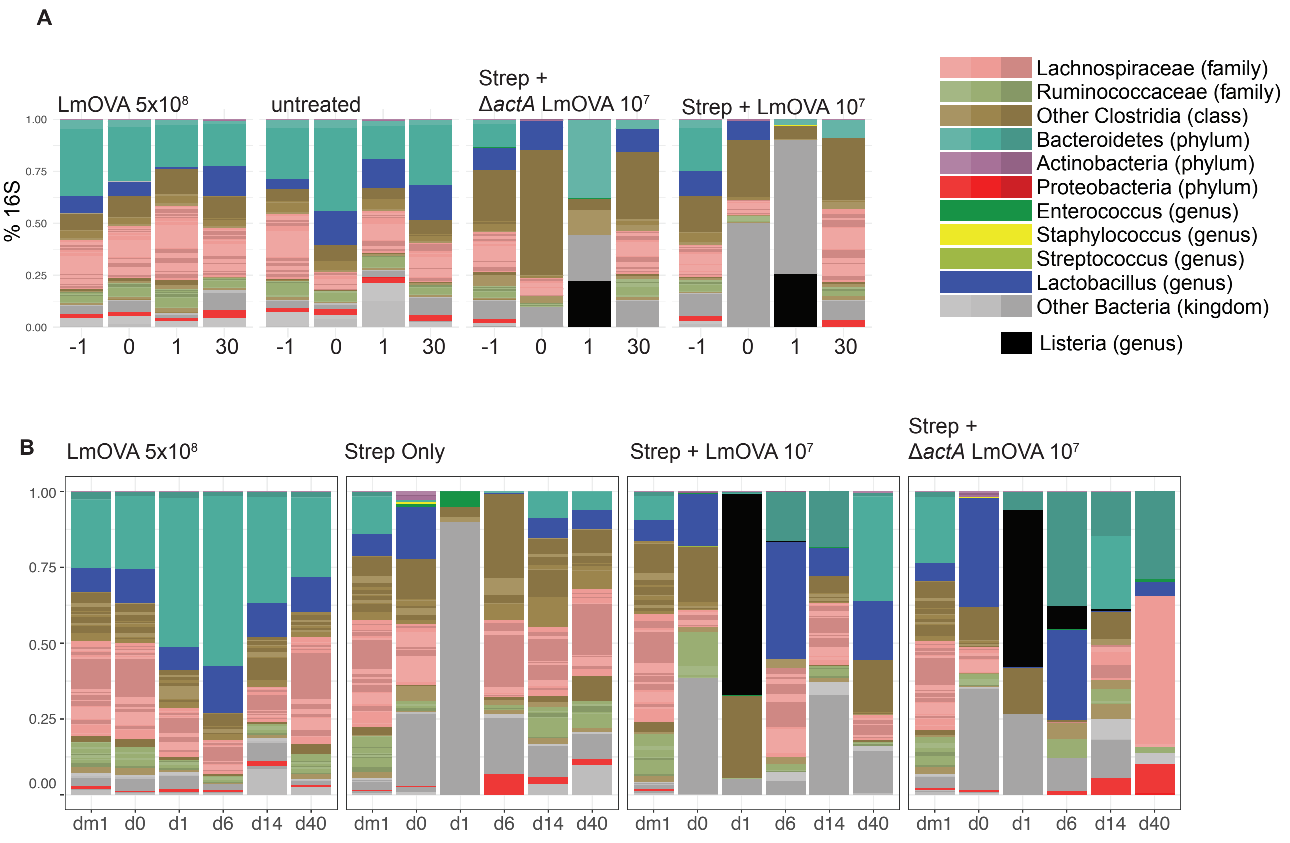

Supplementary Figure 1. Effects of streptomycin treatment and Lm administration on microbiota composition. (A, B) Bargraphs depicting taxa abundance in two replicate experiments of the one shown in Figure 1D-E (each bar represents the average microbiota composition of n=2-5 mice).

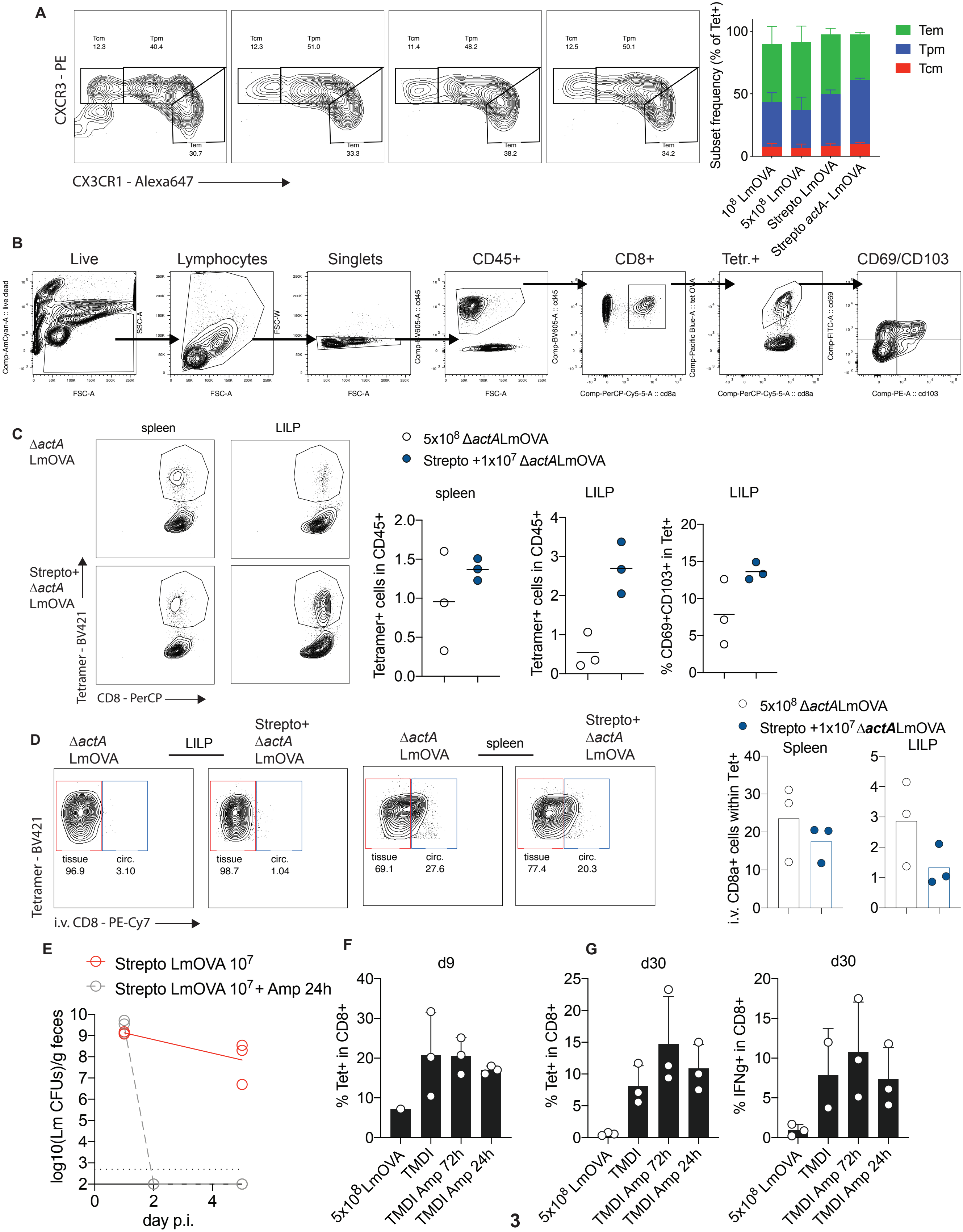

A
Tcm 12.3
Tpm 40.4
Tem 30.7
Tcm 12.3
Tpm 51.0
Tem 33.3
Tcm 11.4
Tpm 48.2
Tem 38.2
Tcm 12.5
Tpm 50.1
Tem 34.2
CXCR3 - PE
CX3CR1 - Alexa647
Subset frequency (% of Tet+)
Tem
Tpm
Tcm
10^8 LmOVA
5x10^8 LmOVA
Strepto LmOVA
Strepto actA- LmOVA
B
Live
Lymphocytes
Singlets
CD45+
CD8+
Tetr.+
CD69/CD103
Comp-AmCyan-A :: live dead
SSC-A
FSC-W
Comp-BV605-A :: cd45
Comp-Pacific Blue-A :: tet OVA
Comp-FITC-A :: cd69
FSC-A
Comp-PerCP-Cy5-5-A :: cd8a
Comp-PE-A :: cd103
C
spleen
LILP
ΔactA LmOVA
Strepto+ ΔactA LmOVA
Tetramer - BV421
CD8 - PerCP
5x10^8 ΔactALmOVA
Strepto +1x10^7 ΔactALmOVA
spleen
LILP
LILP
Tetramer+ cells in CD45+
% CD69+CD103+ in Tet+
D
ΔactA LmOVA
LILP
Strepto+ ΔactA LmOVA
ΔactA LmOVA
spleen
Strepto+ ΔactA LmOVA
tissue 96.9
circ. 3.10
tissue 98.7
circ. 1.04
tissue 69.1
circ. 27.6
tissue 77.4
circ. 20.3
Tetramer - BV421
i.v. CD8 - PE-Cy7
5x10^8 ΔactALmOVA
Strepto +1x10^7 ΔactALmOVA
Spleen
LILP
i.v. CD8a+ cells within Tet+
E
Strepto LmOVA 10^7
Strepto LmOVA 10^7 + Amp 24h
log10(Lm CFUs)/g feces
day p.i.
F
d9
% Tet+ in CD8+
5x10^8 LmOVA
TMDI
TMDI Amp 72h
TMDI Amp 24h
G
d30
% Tet+ in CD8+
d30
% IFNg+ in CD8+

Supplementary Figure 2. Phenotype, tissue residency and kinetics of accumulation of CD8+ T cells generated upon streptomycin-pretreatment. (A) Representative FACS plots and bargraphs depicting the phenotype of circulating tetramer-positive CD8+ T cells using the approach described by Gerlach and collaborators 15 (n=3, shown are means ± SEM). (B) Gating strategy for identification and characterization of OVA-specific CD8 T cells. (C) Representative FACS plots, quantification and CD69/CD103 phenotype of splenic and LILP Tetramer+ CD8 T+ cells generated by streptomycin pre-treatment and gavage with the attenuated ΔactA Lm strain at d9 post inoculum (n=3, shown are means). (D) Mice were immunized following streptomycin treatment using the attenuated ΔactA Lm strain and sacrificed at d9 p.i. Intravascular staining of CD8+ T cells was performed to determine the fraction of circulating contaminants within the tetramer+ CD8+ T cell pool detected in the depicted tissues. Shown are representative FACS plots and total quantifications (n=3, shown are means). (E) Mice were administered streptomycin and after one day were gavaged with LmOVA. 24h later mice were treated with ampicillin (oral gavage + ad libitum drinking water); fecal LmOVA CFUs were enumerated at d1, 2 and 5 (n=2-3, one representative of 2 experiments; shown are geometric means). (F, G) Relative expansion of tetramer+ CD8+ T cells in mice administered LmOVA or streptomycin + LmOVA and then ampicillin 24 or 72h post-inoculum (see Methods section). Tetramer staining of the LILP was performed either on d9 (F) or d30 (G) post inoculum. On d30, cells were also re-stimulated ex vivo with cognate peptide in the presence of BFA and assessed for IFNγ production (n=2-4, shown are means ± SD, experiments shown

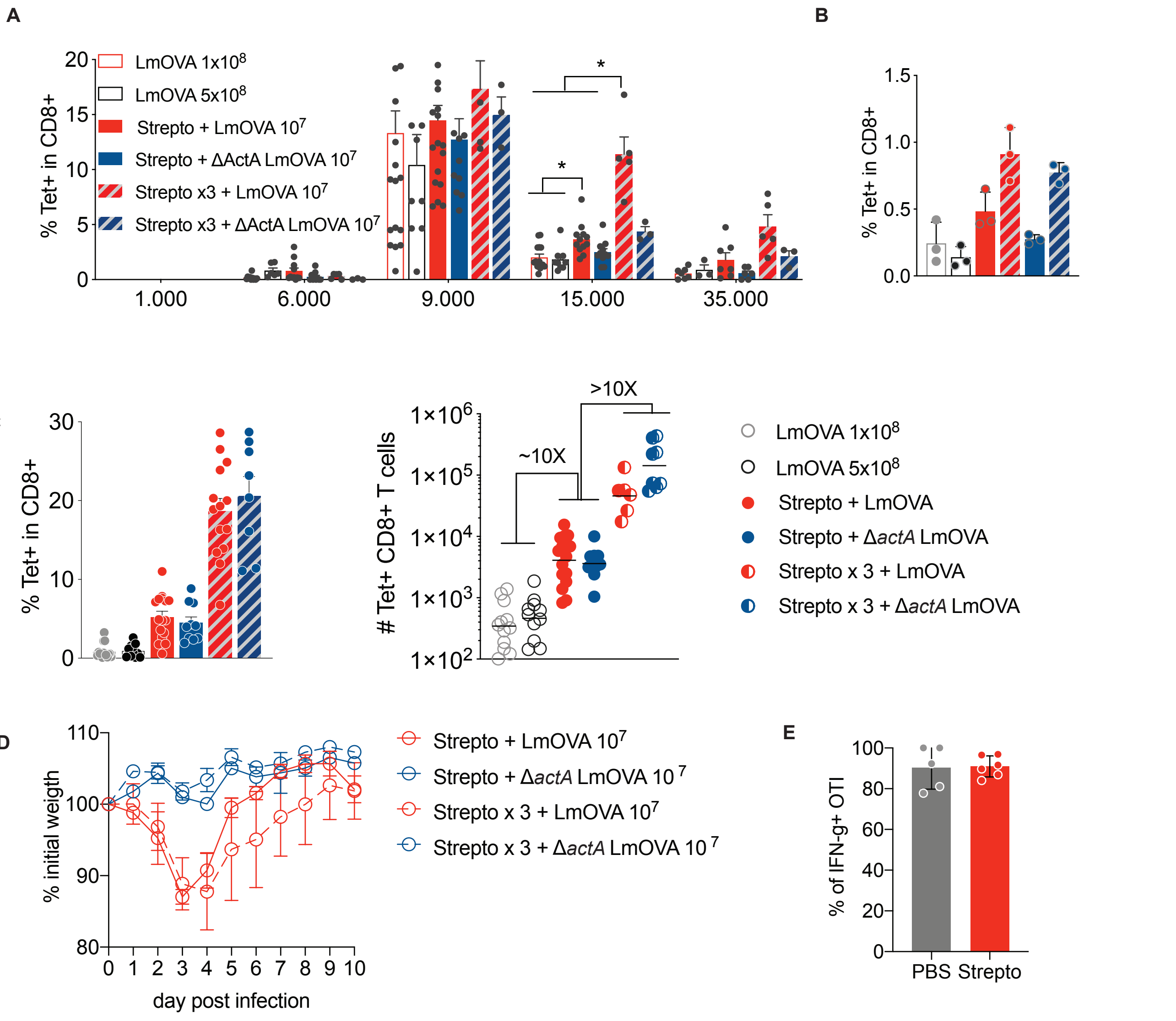

Supplementary Figure 3. Effect of single or repeated streptomycin treatments on number and functions of antigen-specific CD8+ T cells. (A) Kinetics of expansion/contraction of tetramer specific endogenous T cells in the blood of mice immunized as indicated (each time point represents the mean of a minimum of n=3 x 1 experiment, to a maximum of n=20 across 6 experiments, shown are means ± SEM, Two-way ANOVA with multiple comparisons). (B) Percentage of tetramer+ CD8+ T cells in the spleen of animals immunized as depicted at day 50 p.i. (n=3, shown are means ± SD, One-way ANOVA with multiple comparisons). (C) Cumulative data showing % and absolute numbers of tetramer-positive CD8+ T cells in the LILP 35-50 days post infection in all performed experiments (left: n=15, 11, 16, 10, 14, 8 from 5, 4, 5, 4, 4, 2 experiments respectively; right: n= 12, 11, 16, 10 , 6, 8 from 4, 4, 5, 4, 2, 2 independent experiments, respectively; shown are means ± SEM). (D) Weight loss curves for animals treated as depicted (n=2-4 mice per group, shown are means ± SD). (E) Percentage of IFN-Y producing among LILP-resident OTI Trm generated by TMDI of OTI-transferred mice ($1.5x10^5$ cells/mouse), at 35-50 days post infection, re-stimulated ex vivo with cognate peptide in the presence of Brefeldin-A (n= 5-6 from 3 independent experiments, means ± SD).

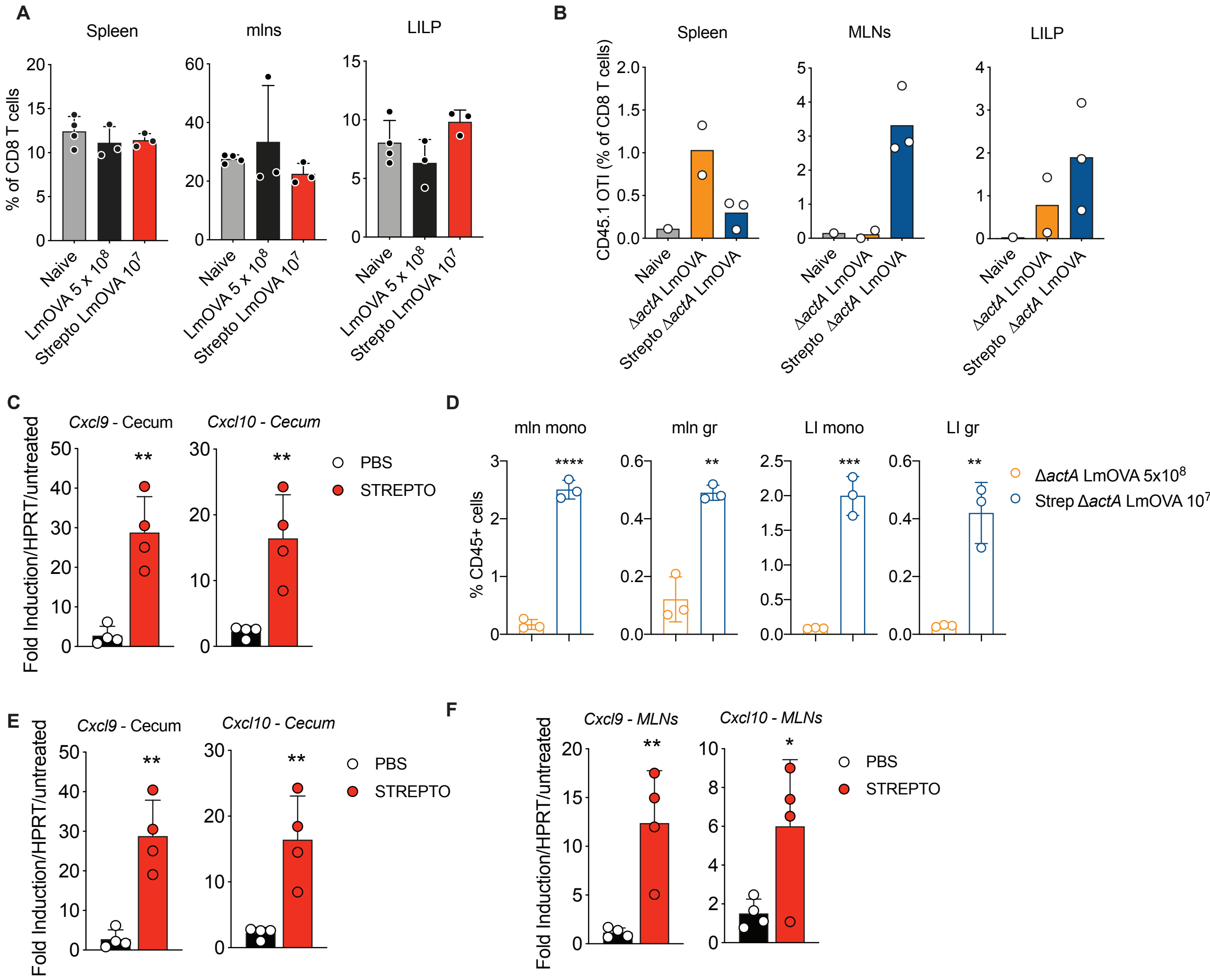

Supplementary Figure 4. Characterization of TMDI using ΔactA LmOVA. (A) Mice receiving 1.5x105 OTI cells were subjected to the depicted immunization approaches, and sacrificed at d4 post infection. Shown are numbers of total CD8+ T cells in the depicted organs (n=3, 1 of 2 experiment shown, means ± SD). (B) OTI accumulation in the depicted organs at d4 following TMDI with ΔactA LmOVA (n=3). (C) qPCR for the depicted cytokines performed on the LI tissue of mice immunized with TMDI using ΔactA LmOVA, at d3 p.i. (n=4, shown are means ± SDEM). (D) Accumulation of granulocytes and monocytes in the MLNs and LILP of mice immunized with TMDI using ΔactA LmOVA, at d2 p.i. (n=3, shown are means ± SD) (E, F) qPCR for the depicted chemokines performed on the cecum or MLN of mice immunized with TMDI using ΔactA LmOVA, at d3 p.i. (n=4, shown are means ± SD) (C-F: data were analyzed with unpaired two-tailed t-test; for all analyses: *=p<0.05, **=p<0.01, ***=p<0.001, ****=p<0.0001).

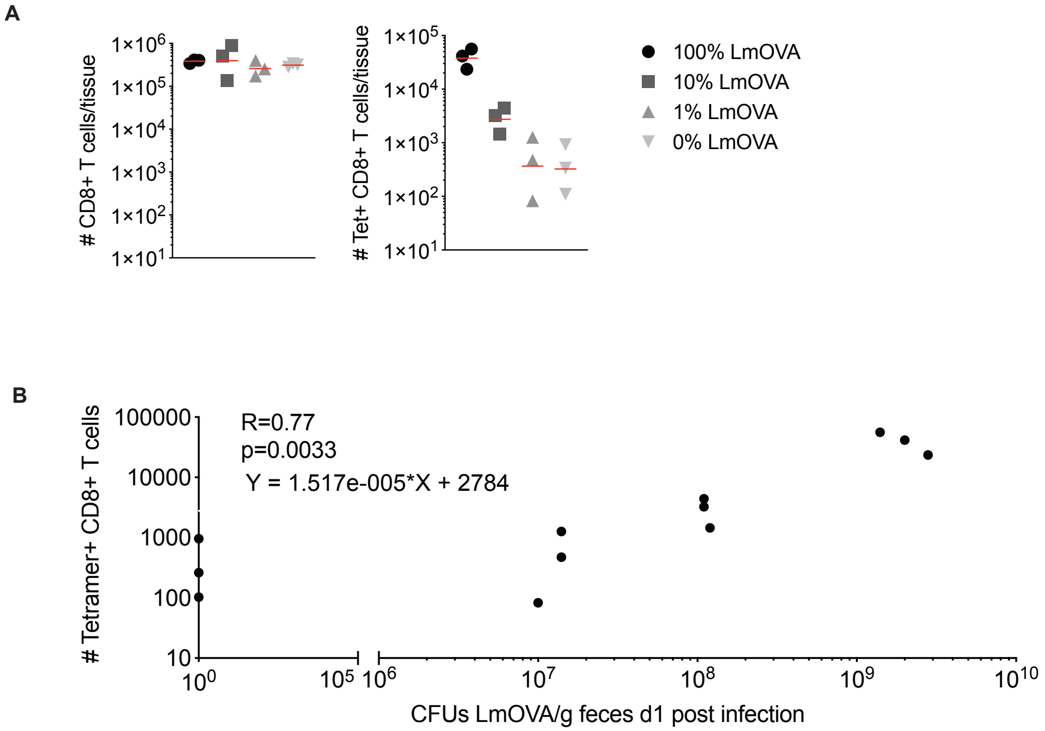

Supplementary Figure 5. Effects of antigen availability on the development of antigen-specific LILP-resident memory CD8+ T cells. (A) Numbers of total CD8+ and SIINFEKL-tetramer+ T cells in the LILP of animals immunized as described in Figure 5 B-H and assessed at day 30 post infection (n=3, shown are means and geometric means). (B) Pearson correlation between absolute numbers of LILP-resident tetramer+ CD8 T cells and LmOVA CFUs/g of feces on day one post inoculum for the experiment depicted in (A) (the R coefficient and two-tailed pvalue are indicated). The results of a linear regression analysis are also indicated. (n=3 per group, total n=12).

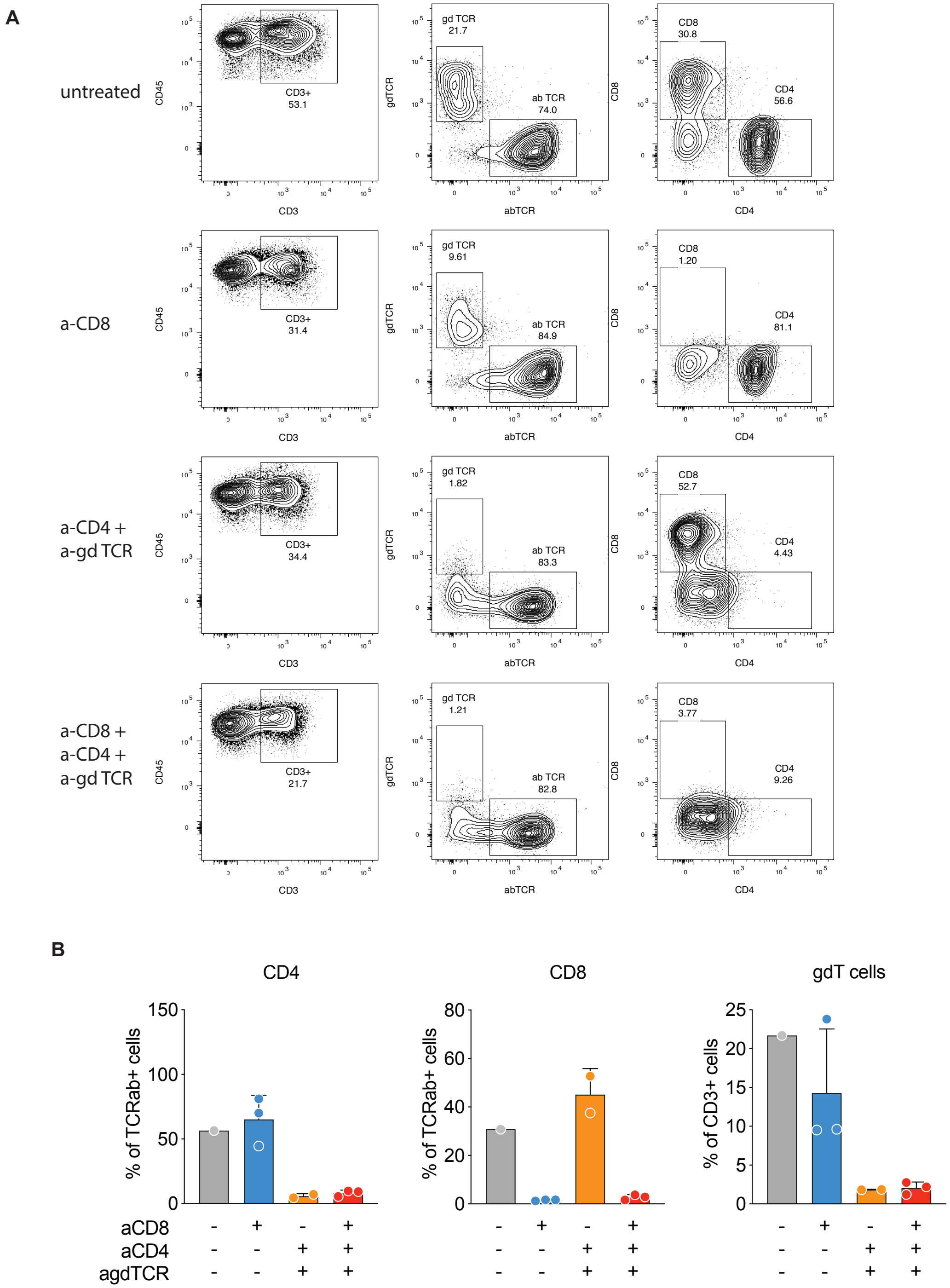

Supplementary Figure 6. Efficiency of antibody-mediated T cell depletion. (A) Representative FACS plots and (B) relative percentages of T cell subsets following in vivo depletion utilizing the indicated antibodies (n=3 per group, shown are means ± SD).
