# Supplementary material for: Enhancing mucosal immunity by transient microbiota depletion
Source: Nat Commun. 2020 Sep 8;11:4475. doi: 10.1038/s41467-020-18248-4 (PMC7479140; doi:10.1038/s41467-020-18248-4)
Supplement: Supplementary file 3 — Reporting Summary [file 41467_2020_18248_MOESM3_ESM.pdf]

nature research

Corresponding author(s): Simone Becattini

Last updated by author(s): Jul 23, 2020

Reporting Summary

Nature Research wishes to improve the reproducibility of the work that we publish. This form provides structure for consistency and transparency in reporting. For further information on Nature Research policies, see our Editorial Policies and the Editorial Policy Checklist.

Statistics

For all statistical analyses, confirm that the following items are present in the figure legend, table legend, main text, or Methods section.

n/a	Confirmed	
☐	☒	The exact sample size (*n*) for each experimental group/condition, given as a discrete number and unit of measurement
☐	☒	A statement on whether measurements were taken from distinct samples or whether the same sample was measured repeatedly
☐	☒	The statistical test(s) used AND whether they are one- or two-sided<br>*Only common tests should be described solely by name; describe more complex techniques in the Methods section.*
☐	☒	A description of all covariates tested
☐	☒	A description of any assumptions or corrections, such as tests of normality and adjustment for multiple comparisons
☐	☒	A full description of the statistical parameters including central tendency (e.g. means) or other basic estimates (e.g. regression coefficient) AND variation (e.g. standard deviation) or associated estimates of uncertainty (e.g. confidence intervals)
☒	☐	For null hypothesis testing, the test statistic (e.g. *F*, *t*, *r*) with confidence intervals, effect sizes, degrees of freedom and *P* value noted<br>*Give P values as exact values whenever suitable.*
☒	☐	For Bayesian analysis, information on the choice of priors and Markov chain Monte Carlo settings
☒	☐	For hierarchical and complex designs, identification of the appropriate level for tests and full reporting of outcomes
☐	☒	Estimates of effect sizes (e.g. Cohen's *d*, Pearson's *r*), indicating how they were calculated

Our web collection on statistics for biologists contains articles on many of the points above.

Software and code

Policy information about availability of computer code

Data collection	Diva Software v.8
Data analysis	FlowJo v.10, R (v.4.0.0) and GraphPad Prism (version 8.4.2) software packages were used for statistical analyses. The DADA2 pipeline was used to analyze 16s rRNA gene sequencing. Custom R codes were generated to analyze 16s rRNA gene data (Unifrac Distance and PCoA shown in Figure 1 and Supplementary Figure 1) and will be available upon request.

For manuscripts utilizing custom algorithms or software that are central to the research but not yet described in published literature, software must be made available to editors and reviewers. We strongly encourage code deposition in a community repository (e.g. GitHub). See the Nature Research guidelines for submitting code & software for further information.

Data

Policy information about availability of data

All manuscripts must include a data availability statement. This statement should provide the following information, where applicable:

- Accession codes, unique identifiers, or web links for publicly available datasets
- A list of figures that have associated raw data
- A description of any restrictions on data availability

Microbiome sequencing data are available from the Bioproject with the accession number PRJNA634963. All other raw data are included in the Source Data file.

Field-specific reporting

Please select the one below that is the best fit for your research. If you are not sure, read the appropriate sections before making your selection.

☒ Life sciences ☐ Behavioural & social sciences ☐ Ecological, evolutionary & environmental sciences

For a reference copy of the document with all sections, see nature.com/documents/nr-reporting-summary-flat.pdf

Life sciences study design

All studies must disclose on these points even when the disclosure is negative.

Sample size	No sample size calculation was performed.
Data exclusions	No data were excluded from analyses.
Replication	Experiments were mostly repeated at least two times, with the exception of the experiments shown in Figure 6B for which some groups were tested only once, and Supplementary Figure 2, 4, 5 which mostly represent confirmation of the results displayed in main figures using a different Listeria strain (delta-ActA Lm OVA). All attempts of replication of results were successful.
Randomization	Microbiota randomization was performed prior to administering TMDI by assigning to each experimental group 1 or multiple mice from each utilized cage.
Blinding	No blinding was performed.

Reporting for specific materials, systems and methods

We require information from authors about some types of materials, experimental systems and methods used in many studies. Here, indicate whether each material, system or method listed is relevant to your study. If you are not sure if a list item applies to your research, read the appropriate section before selecting a response.

Materials & experimental systems

n/a	Involved in the study	
☐	☒	Antibodies
☒	☐	Eukaryotic cell lines
☒	☐	Palaeontology and archaeology
☐	☒	Animals and other organisms
☒	☐	Human research participants
☒	☐	Clinical data
☒	☐	Dual use research of concern

Methods

n/a	Involved in the study	
☒	☐	ChIP-seq
☐	☒	Flow cytometry
☒	☐	MRI-based neuroimaging

Antibodies

Antibodies used	Fc blocking (BD #553142), CD4 (clone RM4-4, BD), CD45 (clone 30-F11 eBioscience), CD45.1 (clone A20, BD), CD45.2 (clone 104, BD) CD8a (clone 53-6.7, eBioscience or BD), CD8b (clone YTS156.7.7, BioLegend), CD11b (clone RM2817, Thermo Fisher), Ly6c (clone AL-21, BD), CD3ε (clone 145-2C11, BD), CD19 (clone 1D3, BD), CD90.2 (53-2.1, BD), CD127 (clone A7R34, BD), CX3CR1 (clone SA011F11, BioLegend), CXCR3 (clone CXCR3-173, Biolegend), CD103 (clone 2E7, eBioscience or Biolegend), CD69 (clone H1.2F3, BD), TCR-β (clone H57-597, BD, TCR-γδ (clone eBio-GL3, eBioscience), IFN-g (clone XMG1.2, BD), TNF-α (clone MP6-XT22, BD).
Validation	All antibodies used were previously validated by the manufacturer and/or in publications.

Animals and other organisms

Policy information about studies involving animals; ARRIVE guidelines recommended for reporting animal research

Laboratory animals	All mice utilized in this study were on the C57Bl/6 background. WT animals were 6-9 weeks-old females purchased from Jackson Laboratories. CD45.1 OTI, CCR2 KO and CXCR3 KO mice were bred at the MSKCC animal facility.
Wild animals	N/A
Field-collected samples	N/A
Ethics oversight	Experiments were performed in compliance with Memorial Sloan-Kettering Cancer Center institutional guidelines and approved by the institution’s Institutional Animal Care and Use Committee.

Note that full information on the approval of the study protocol must also be provided in the manuscript.

Flow Cytometry

Plots

Confirm that:

☒ The axis labels state the marker and fluorochrome used (e.g. CD4-FITC).

☒ The axis scales are clearly visible. Include numbers along axes only for bottom left plot of group (a 'group' is an analysis of identical markers).

☒ All plots are contour plots with outliers or pseudocolor plots.

☒ A numerical value for number of cells or percentage (with statistics) is provided.

Methodology

Sample preparation	Blood was obtained by tail bleeding. Red blood cell lysis was performed by 3 consecutive incubations in RBC lysis buffer (0.15 M NH_4Cl , 1 mM $NaHCO_3$ in dH_2O) for 5'.<br>Lymphocytes were isolated from the mLNs by mechanical disruption through 100-µm cell strainers.<br>Splenocytes were isolated by mechanical disruption of the spleen through s 100-µm cell strainer, followed by RBS lysis (1 RBC lysis buffer incubation for 2 minutes, followed by wash in complete medium).<br>Single-cell suspensions were obtained from the large intestine lamina propria by longitudinally cutting the tissue and then washing out content in PBS. Intestinal tissues were incubated at 37°C under gentle agitation in stripping buffer [PBS, 5 mM EDTA, 1 mM dithiothreitol, 4% fetal calf serum, and penicillin/streptomycin (10 µg/ml)] for 30 min. The remaining tissue was digested with collagenase IV (1.5 mg/ml; 500 U/ml) and deoxyribonuclease (20 µg/ml) in complete medium [Dulbecco's modified Eagle's medium supplemented with 10% fetal bovine serum, penicillin/streptomycin (10 µg/ml), gentamicin (50 µg/ml), 10 mM Hepes, 0.5 mM β-mercaptoethanol, and L-glutamine (20 µg/ml)] for 30 min at 37°C under gentle agitation.<br>Supernatants containing the Lp fraction were passed through a 100-µm cell strainers and resuspended in 40% Percoll. Samples were then centrifuged for 20 min at 600g.<br>Cells from all of the above sources were washed and resuspended in PBS for viability staining. FACS staining was performed in FACS buffer (PBS + 2% FCS).
Instrument	BD LSR II.
Software	FACS Diva v8.0.1
Cell population abundance	No sorting was performed. Purity of OTI cells following bead-based negative enrichment on OTI splenocytes was performed by CD8 staining on the total recovered population.
Gating strategy	Death cells were gated out based on live/dead staining. Sequential gating was then performed based on: FSC/SSC, FSC-W/FSC-H (singlets), CD45+, CD8+, CD8/Tetramer+, and finally CD69/CD103. For IC staining, gating included live/dead, CD45, CD8, and IFNg/TNFa. For OTI transfer experiments, cells were first gated on live/dead, total CD45 positive (CD45.1+ and CD45.2+), CD8+, and then subsetted for CD45.1 and CD45.2.

☒ Tick this box to confirm that a figure exemplifying the gating strategy is provided in the Supplementary Information.
